# An unusual case of lower limb claudication in a young male: a case report

**DOI:** 10.1590/1677-5449.202500112

**Published:** 2025-08-13

**Authors:** Thilina Gunawardena, Waruni Dissanayaka

**Affiliations:** 1 Teaching Hospital Polonnaruwa, Department of Vascular and Transplant Surgery, Polonnaruwa, Sri Lanka.; 2 Teaching Hospital Polonnaruwa, Department of Pathology, Polonnaruwa, Sri Lanka.

**Keywords:** spontaneous dissection, iliac artery, graft, dissecção espontânea, artéria ilíaca, enxerto

## Abstract

Exertional lower extremity pain due to arterial insufficiency is uncommon in the young. In this case report we present a 34-year-old, previously healthy male with sudden onset left lower limb pain upon walking for 50 meters. He was found to have a spontaneous dissection of his external iliac artery, which is a rare pathology. After successful open revascularization, he made an uneventful recovery.

## Resumo

A dor em membros inferiores desencadeada por esforço físico, decorrente de insuficiência arterial, é pouco frequente em pacientes jovens. Neste relato, descrevemos o caso de um homem de 34 anos, previamente saudável, que apresentou dor súbita no membro inferior esquerdo ao caminhar por 50 metros. Foi constatada uma dissecção espontânea da artéria ilíaca externa, uma patologia rara. Após revascularização aberta bem-sucedida, o paciente se recuperou sem intercorrências.

## INTRODUCTION

Intermittent claudication (IC) is defined as exercise induced extremity pain that is relieved by rest. It is a common presentation of peripheral arterial disease, for which atherosclerosis is the leading predisposing factor, especially in the elderly. Young patients who do not have risk factors for atherosclerosis can occasionally present with lower limb claudication due to causes such as Buerger’s disease, cystic adventitial disease, vasculitis, and arterial entrapment syndromes.^1,2^ Here, we describe a healthy 34-year-old male who presented with acute onset short distance claudication of his left lower limb. The symptoms followed a bout of vigorous flank exercises. During workup, he was found to have an isolated dissection of his distal common iliac artery (CIA) extending along the external iliac artery (EIA) up to the groin. Dissection of an otherwise normal iliac artery without aortic involvement is a rare condition. Due to the lifestyle limiting nature of his symptoms, we replaced his dissected iliac artery with an interposition graft, with an excellent outcome. Informed, written consent was obtained from the patient before publication of the case report and the study was done as per the standards of the institutional ethics committee and the Helsinki declaration.

## CASE REPORT

A 34-year-old male presented to the vascular surgery outpatient clinic with exercise induced left calf pain of 2 weeks’ duration. The pain was typical of IC with onset at around 50 meters. He was a non-smoker and had no significant past medical history. The lower limb symptoms were associated with a poorly defined left iliac fossa and groin pain. On further inquiry, he recalled that the symptom onset was sudden following a vigorous bout of twisting exercises for the flank. He did not engage in any form of contact sport and denied the use of anabolic steroids.

His blood pressure was normal. The abdomen and the groin were soft with no palpable masses. The left femoral pulse was weakly palpable, but popliteal and pedal pulses were absent. On the contralateral limb, good volume dorsalis pedis and posterior tibial pulses were present. The ankle-brachial pressure index (ABPI) at rest for the affected extremity was 0.8, while it was 1 for the contralateral limb.

**Figure 1:**
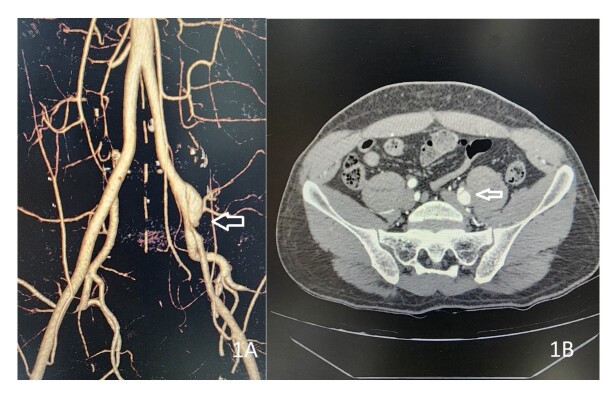
(A) Reconstructed CT image depicting the narrow true lumen of the EIA; (B) Axial CT image depicting the origin of the dissection.

Duplex ultrasound scan (DUS) of the left lower limb revealed a patent arterial tree from the groin up to the foot, but the flow was comparatively poor compared to the right with low velocity biphasic wave patterns in the femoral artery and downstream arteries. Suspecting a suprainguinal arterial pathology, he underwent a computed tomography angiogram (CTA) which depicted a dissection starting from the left distal CIA and extending along the EIA almost up to the inguinal ligament (Figures 1A and 1B). There was ectasia of the CIA at the origin of the dissection flap. The true lumen of the EIA was significantly compromised by the thrombosed false lumen.

**Figure 2:**
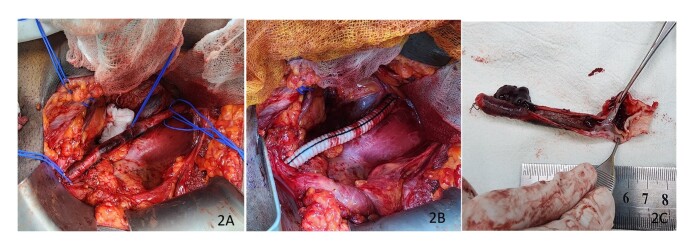
(A) Dissected iliac artery; (B) PTFE interposition graft; (C) Excised specimen with the thrombosed false lumen.

**Figure 3:**
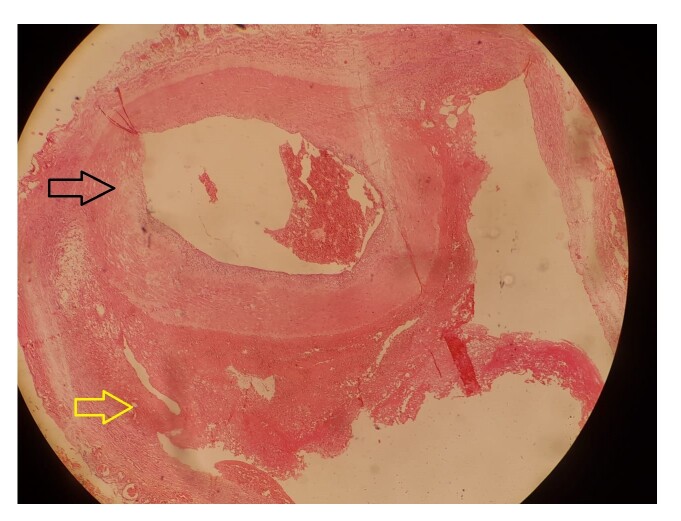
Histology of the specimen (Black arrow: true lumen; Yellow arrow: false lumen).

As the lower limb symptoms were lifestyle limiting, surgical repair was offered to the patient. He had a complete blood count, erythrocyte sedimentation rate, and 2D echocardiogram, which were all within normal limits. Under combined spinal-epidural anesthesia, the left iliac vessels were exposed using an extraperitoneal approach. The CIA, EIA, and internal iliac artery (IIA) were dissected and circumferential control was achieved (Figure 2A). After intravenous administration of 5,000 units of unfractionated heparin, the distal EIA, IIA, and CIA were clamped. The distal CIA and the dissected EIA were excised after ligation of the IIA. Continuity of the CIA and EIA was restored using an 8 mm polyester interposition graft in an end-to-end fashion (Figures 2B and 2C). Left pedal pulses were palpable immediately after releasing the clamps. Histology of the excised specimen confirmed dissection in an otherwise normal artery. (Figure 3) The patient made an uneventful recovery and was discharged on post-operative day 3 with low-dose acetylsalicylic acid. His ABPI on the left improved to 1, and at 6 months follow-up he remains well with palpable pedal pulses, free from claudication.

## DISCUSSION

Isolated, spontaneous dissection of the iliac arteries is rare.^3,4^ Conditions such as atherosclerosis, systemic arterial hypertension, trauma, fibromuscular dysplasia, collagen vascular disorders, and pregnancy have been linked with it.^4^ However, the patient described in this case report had none of these risk factors. Considering the temporal relationship between the vigorous flank exercises and the onset of the symptoms, we believe that his arterial injury was most likely exercise-induced. Aggressive exercise can dramatically increase blood pressure and cardiac output. Arteries exposed to such extreme physiological stress can develop vessel wall injury. This is especially seen at branching points of blood vessels, where there is maximum turbulence.^5-7^ However, dissection of an otherwise healthy artery due to exercise is an extremely rare phenomenon.^6^ Cook and colleagues reported 3 such cases in athletes who were above 40 years of age. All developed dissections of their EIAs after intense exercise.^7^ A different case published by Teh et al. reported spontaneous dissection of a CIA in a 60-year-old cyclist.^6^ Our patient is comparatively younger and shows that this complication can happen in otherwise healthy individuals even in their thirties.

Sudden onset lower limb claudication, abdominal pain, limb threatening Rutherford grade 2 or 3 acute ischemia and hemorrhagic shock from rupture of the injured artery have been reported as presentations of spontaneous iliac artery dissections.^4-8^ The diagnosis is generally confirmed after imaging studies such as DUS and CTA.^6^

Once diagnosed, there is no consensus on the best management strategy for this condition. Conservative management, open surgery, and endovascular stenting have all been used as options.^5^ Intervention is indicated for alleviation of symptoms and to prevent future complications such as arterial rupture and aneurysmal degeneration.^9^ Our patient was young and active with lifestyle limiting claudication, so intervention was offered.

Percutaneous stenting of the dissected iliac artery to seal the entry site of the dissection is an attractive choice due to its minimally invasive nature. The relining of the true lumen with a stent promotes favorable vascular remodeling. This reduces the future risk of rupture and aneurysm formation.^4^ However, the long-term outcomes of iliac stents are not known and there can be in-stent stenoses and stent fractures. These issues should be considered when using a stent in a young patient with a long life expectancy.^10^

In contrast, open surgery with bypass or resection and replacement of the damaged segment of the vessel can be considered a more durable option, albeit with greater procedure-related morbidity. In our patient, there were several reasons for opting for open surgery. First, in Sri Lanka endovascular facilities are available in only a few tertiary care hospitals and off-the-shelf stents of appropriate sizes are rarely available. The patient was in his thirties and the longevity of a stent and the long-term follow-up required to monitor stent patency and its complications would have been an issue. Thus we opted for resection of the dissected segment of the artery with interposition polyester graft placement. To optimize graft patency, we started him on 75 mg of acetylsalicylic acid per day and intend to continue it long-term. This is common practice after vascular grafting.^4^

## CONCLUSIONS

Isolated dissection of the iliac artery without a clear predisposing pathology is a rare presentation. Our case highlights that open surgery in such cases can achieve excellent outcomes.

## References

[1] Cassar K (2006). Intermittent claudication. BMJ.

[2] Hallett JW, Greenwood LH, Robison JG (1985). Lower extremity arterial disease in young adults. A systematic approach to early diagnosis. Ann Surg.

[3] Lu AK, Baldwin J, Hans SS (2023). Acute limb ischemia secondary to external iliac and common femoral artery dissection in a body builder. J Vasc Surg Cases Innov Tech.

[4] Tanda E, Genadiev GG, Zappadu S, Donno G, Camparini S (2021). Spontaneous isolated dissection of iliac artery treated with endovascular repair: a case report. Vasc Spec Int.

[5] Yamanaka Y, Yoshida T, Nagaoka E (2017). Bilateral external iliac artery dissection in a middle-aged male athlete. Ann Vasc Dis.

[6] Teh LG, Sieunarine K, Van Schie G, Vasudevan T (2003). Spontaneous common iliac artery dissection after exercise. J Endovasc Ther.

[7] Cook PS, Erdoes LS, Selzer PM, Rivera FJ, Palmaz JC (1995). Dissection of the external iliac artery in highly trained athletes. J Vasc Surg.

[8] Bosio PM, Barry-Kinsella C, O’Keane C, O’Malley K (1998). Spontaneous iliac artery dissection in a healthy postpartum woman. J Obstet Gynaecol.

[9] Kwon SH, Oh JH (2006). Successful interventional treatment of a spontaneous right common iliac artery dissection extending retrogradely into the left external iliac artery. J Vasc Interv Radiol.

[10] Kwak HS, Han YM, Chung GH, Yu HC, Jeong YJ (2006). Isolated spontaneous dissection of the common iliac artery: percutaneous stent placement in two patients. Cardiovasc Intervent Radiol.

